# Histamine up-regulates fibroblast growth factor receptor 1 and increases FOXP2 neurons in cultured neural precursors by histamine type 1 receptor activation: conceivable role of histamine in neurogenesis during cortical development *in vivo*

**DOI:** 10.1186/1749-8104-8-4

**Published:** 2013-03-07

**Authors:** Anayansi Molina-Hernández, Griselda Rodríguez-Martínez, Itzel Escobedo-Ávila, Iván Velasco

**Affiliations:** 1 Instituto de Fisiología Celular - Neurociencias, Universidad Nacional Autónoma de México, México, D.F. 04510, México; 2 Departamento de Biología Celular, Instituto Nacional de Perinatología “Isidro Espinosa de los Reyes”, Montes Urales 800, Colonia Lomas de Virreyes, México, D.F., C.P. 11000, México

**Keywords:** Neural stem cells, H_1_R, Clonal analysis, FGF receptors, Cerebral cortex, FOXP2

## Abstract

**Background:**

During rat development, histamine (HA) is one of the first neuroactive molecules to appear in the brain, reaching its maximal value at embryonic day 14, a period when neurogenesis of deep layers is occurring in the cerebral cortex, suggesting a role of this amine in neuronal specification. We previously reported, using high-density cerebrocortical neural precursor cultures, that micromolar HA enhanced the effect of fibroblast growth factor (FGF)-2 on proliferation, and that HA increased neuronal differentiation, due to HA type 1 receptor (H_1_R) activation.

**Results:**

Clonal experiments performed here showed that HA decreased colony size and caused a significant increase in the percentage of clones containing mature neurons through H_1_R stimulation. In proliferating precursors, we studied whether HA activates G protein-coupled receptors linked to intracellular calcium increases. Neural cells presented an increase in cytoplasmic calcium even in the absence of extracellular calcium, a response mediated by H_1_R. Since FGF receptors (FGFRs) are known to be key players in cell proliferation and differentiation, we determined whether HA modifies the expression of FGFRs1-4 by using RT-PCR. An important transcriptional increase in FGFR1 was elicited after H_1_R activation. We also tested whether HA promotes differentiation specifically to neurons with molecular markers of different cortical layers by immunocytochemistry. HA caused significant increases in cells expressing the deep layer neuronal marker FOXP2; this induction of FOXP2-positive neurons elicited by HA was blocked by the H_1_R antagonist chlorpheniramine *in vitro*. Finally, we found a notable decrease in FOXP2+ cortical neurons *in vivo*, when chlorpheniramine was infused in the cerebral ventricles through intrauterine injection.

**Conclusion:**

These results show that HA, by activating H_1_R, has a neurogenic effect in clonal conditions and suggest that intracellular calcium elevation and transcriptional up-regulation of FGFR1 participate in HA-induced neuronal differentiation to FOXP2 cells *in vitro*; furthermore, H_1_R blockade *in vivo* resulted in decreased cortical FOXP2+ neurons.

## Background

The mammalian neocortex comprises six layers, each containing neurons with its own morphology, functional properties and connections as well as time of origin. Neocortex formation during development is generated in an inside-out pattern, with the oldest neurons (layer VI) closest and the youngest neurons (layers II/III) farthest from their birthplace near the ventricle [1-4]. The region where migration stops is defined by a layer of specialized pioneer neurons called Cajal-Retzius cells, which migrate tangentially early during development [5].

After development, the deepest layers of cortical neurons (layers V and VI) contain neurons that interconnect areas of the cortex or projection neurons reaching subcortical structures. Layer IV is the layer where most extracortical inputs arrive, particularly from the sensory thalamus, whereas the superficial layers are composed mainly of local circuits that form reciprocal connections with the deep layers [6]. Neurons within the cortex are born in a precise timing and this is essential for generating the final complex laminar cytoarchitecture, in which it is possible to identify specific layer markers such as reelin and p73 (layer I), stab2 and Cux1 (layers II/III and IV), Tbr1, CTIP2, Sox5, Tle4, FOXP2, ER81, OTX1 and Stab2 (layers V and VIa) and, finally, Tbr1 and CTIP2 for layer VIb [7,8].

During rat development, histamine (HA) is one of the first neuroactive molecules to be detected in the central nervous system (CNS), being present as early as embryonic day (E)12 and reaching its maximum value at E14 to E16 in the prosencephalic area [9], a five-fold higher value relative to adult brain levels. Between E14 and E18, fibers from the transient histaminergic neurons can be detected in the mesencephalon, passing through the ventral tegmental area and within the medial forebrain bundle and the optic tract, to reach the frontal and the parietal cortices at E15, earlier than other monoaminergic systems [9-13]; this coincides with the period in which neuronal differentiation is occurring in the cerebral cortex [14]. This rise in HA, together with mRNA expression for HA receptors type 1 (H_1_R) and type 2 (H_2_R) in the developing CNS suggests a role of HA during brain development [15].

The *in vitro* effects of HA on proliferation and neuronal differentiation of cerebral cortex neural precursor cells (NPC) were studied by our group. We showed that 100 μM HA increases cell proliferation mainly through H_2_R activation without causing premature differentiation in the presence of fibroblast growth factor (FGF)-2 [16]. We have recently reported that HA is required in the proliferative phase (+FGF-2) of NPC to induce neurogenesis [17]. After FGF-2 withdrawal, HA augmented neuronal differentiation by H_1_R stimulation [16]. H_1_R is a G protein-coupled receptor, which after activation produces inositol triphosphate (IP_3_) and diacylglycerol, that in turn promote an increase in [Ca^2+^]_i_ due to activation of IP_3_ receptors in the endoplasmic reticulum, and the activation of protein kinase C [18]. Calcium release from intracellular stores into the cytosol is a critical component during ontogenesis and contributes particularly to the formation and maintenance of dendritic structures [19,20].

In this report we studied whether HA-induced neurogenesis was present at the single-cell level by clonal analysis. In proliferating NPC, HA induced calcium elevations mediated by H_1_R activation. FGF receptors (FGFRs) transcripts were up-regulated by HA in the presence of FGF-2, with FGFR1 presenting a sustained elevation after two hours. Cultured NPC readily differentiated to neurons that express the deep cortical layers marker FOXP2 after HA treatment. Antagonism of H_1_R *in vivo* during cortical development resulted in decreased immuno-reactivity to β-tubulin III and FOXP2.

## Results

We have previously reported that NPC, before and after differentiation, express H_1_R and H_2_R mRNA (by RT-PCR) and their corresponding proteins (by immunoblot). We analyzed H_1_R and H_2_R expression and its regulation by HA at the cellular level, using specific antibodies for these histaminergic receptors. Additional file 1 shows that H_1_R and H_2_R are present in 81 ± 1.8% and 92 ± 2.0% of passage (P) 2 proliferating NPC, respectively. Cultures treated with 100 μM HA for 4 days showed very similar proportions of H_1_R- (83 ± 1.0%) and H_2_R-positive cells (94 ± 1.4%).

### Neuronal differentiation is promoted by histamine in clonally-derived colonies

**Figure 1:**
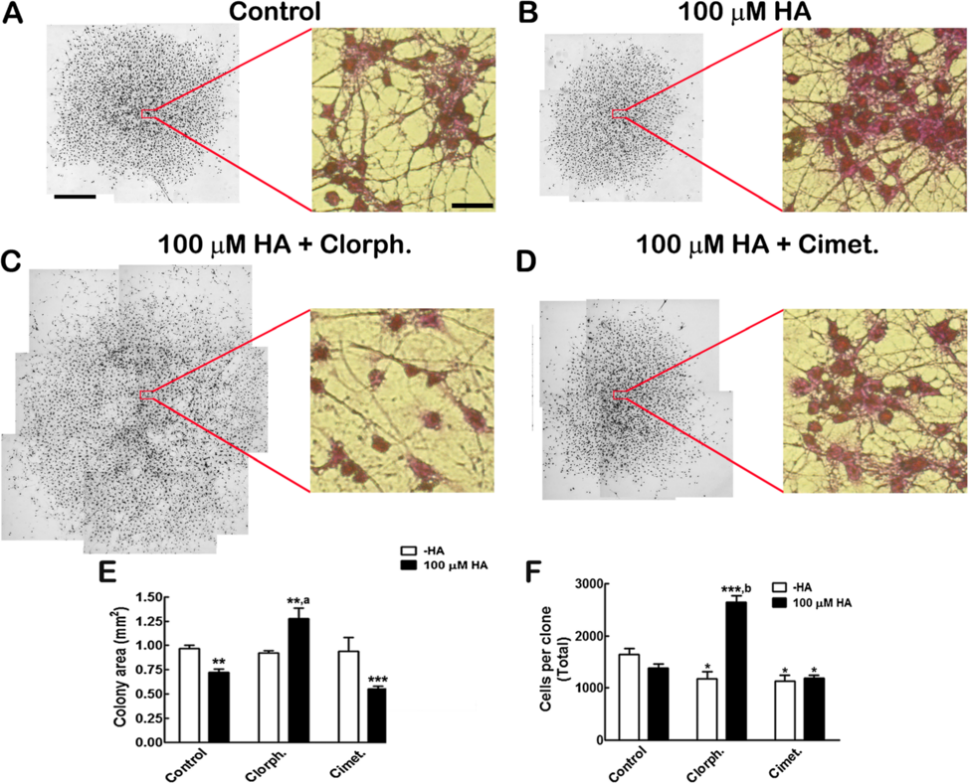
**HA significantly decreases clonal colony size by H**_**1**_**R activation. **Crystal violet-stained clones were used to measure colony area and the number of cells. **(A-D) **Representative composite pictures of colonies with the indicated treatment. The insets show a magnification of the center of each colony. Note that cell size seems uniform, but in 100 μM HA + chlorpheniramine (Chlorph.) the cells are more separated from each other. Scale bar = 250 μm for the low magnification and 100 μm for the insets. **(E) **Areas (mm^2^) quantified from crystal violet-stained clones. HA reduces the colony’s area in a significant manner with respect to control cultures; this effect was prevented by the H_1_R antagonist chlorpheniramine (Chlorph.), but not by cimetidine (Cimet.). **P <0.01 and ***P <0.001 versus Control; ^*a*^*P <0.01 *versus 100 μM HA. **(F) **Cell counting from the stained clones, showing a non-significant decrease in the total number of cells after HA treatment and a significant increase with HA + Chlorph. *P <0.05 versus Control; ***P <0.001 versus Control; ^*b*^*P *<0.001 versus clorpherinamine-HA. Results are the mean ± SEM from three independent experiments made in duplicate. HA, histamine; H_1_R, histamine receptor type 1; SEM, standard error of the mean.

HA increases the proportion of differentiated neurons in cortical NPC cultures, an effect mediated by H_1_R activation [16]. We performed experiments at clonal density, in which HA was present all along proliferation and differentiation phases, to establish if this neurogenic effect was also present in colonies arising from a single cell. Isolated individual cells were identified and allowed to proliferate in the presence of FGF-2 for eight days, followed by FGF withdrawal for six additional days to induce differentiation. Since we observed apparent changes in the size of colonies between the control and HA condition, we measured the area and total cell numbers per clonal colony after crystal violet staining (Figures 1A-D). HA caused a significant 26% decrease in colony area (Figure 1E) and also a 16% non-significant reduction in cell number (Figure 1F). Co-incubation of HA with the H_1_R antagonist chlorpheniramine caused a significant increase in both colony area and cell number, relative to control and also to 100 μM HA. Incubation of HA with the H_2_R antagonist cimetidine produced colonies significantly smaller than controls; cell number per colony was also significantly decreased by cimetidine, regardless of HA presence, relative to the control value.

**Figure 2:**
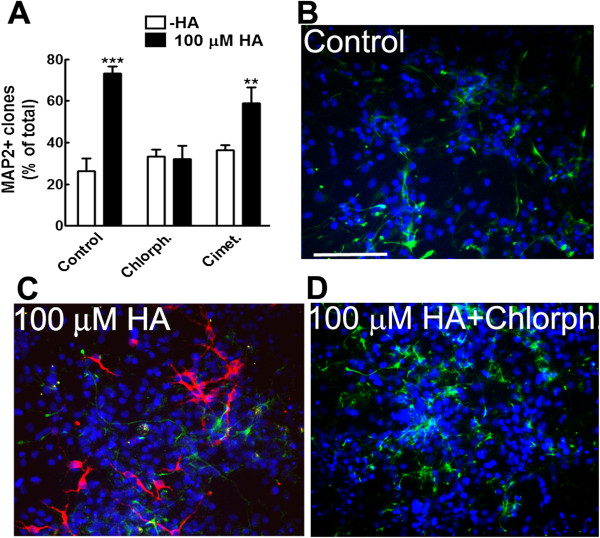
**HA activates H**_**1**_**R to increase the number of clones with differentiated neurons. (A)** Significant increase in the number of colonies with microtubule associated protein 2 (MAP2)+ neurons promoted by HA and its prevention by chlorpheniramine (Chlorph.) **P <0.01 and ***P <0.001 versus Control. **(B-D) **Representative micrographs of clonal colonies after immunocytochemical detection of mature neurons (MAP2+; red), astrocytes (GFAP+; green) and nuclei (blue) in Control **(B)**, HA-treated **(C) **and HA + chlorpheniramine (Chlorph.)-treated **(D) **cells are shown. Scale bar = 100 μm. GFAP, glial fibrillary acidic protein; HA, histamine; H_1_R, histamine receptor type 1.

We next analyzed cell fate in these clonal colonies. Mature neurons (MAP2+) and astrocytes (GFAP+) were detected by immunofluorescence to score the proportion of colonies that contained mature neurons. HA increased 2.8-fold the number of colonies with neurons after differentiation (Figure 2); the HA effect was completely prevented when cultures were co-treated with chlorpheniramine, but was insensitive to cimetidine, since no significant difference was found between HA and HA + cimetidine conditions. Cultures treated with the H_1_R or the H_2_R antagonist without HA, did not show significant changes. Concerning astrocytic differentiation, GFAP+ cells were detected in all clonal colonies.

### Histamine causes an increase of intracellular calcium dependent on activation of H_1_R in undifferentiated cells

**Figure 3:**
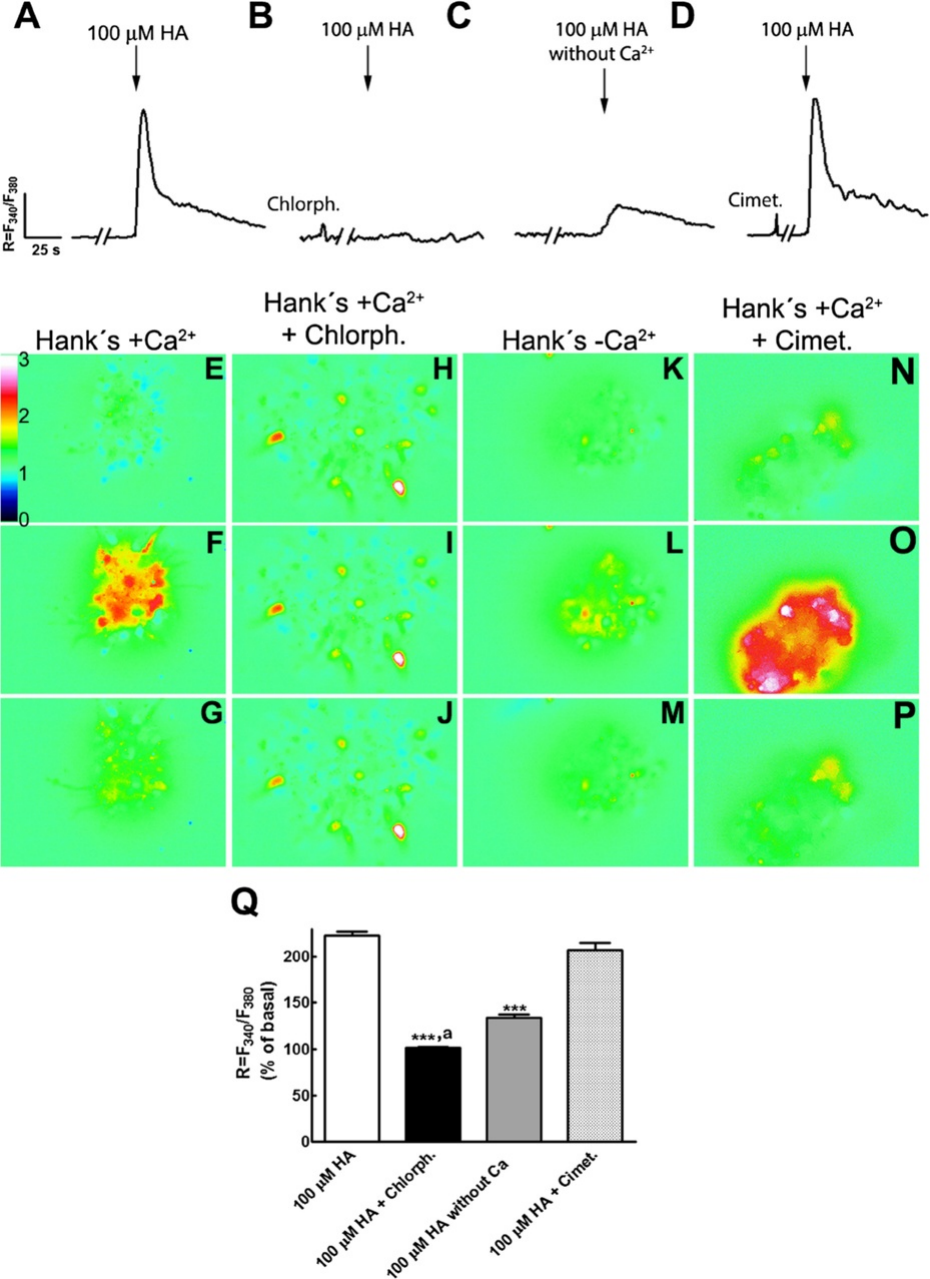
**Increase of intracellular calcium induced by HA through activation of H**_**1**_**R. (A-D) **Representative trace from responsive NPC to 100 μM HA, showing the average ratio (R) of fluorescence of Fura-2 alternating excitation between 340 nm (F_340_) and 380 nm (F_380_) as a measure of [Ca^2+^]_i_. HA addition elicited a clear increase **(A) **that was blocked by co-incubation of HA with the H_1_R antagonist chlorpheniramine (Chlorph.) **(B)**, **(C) **HA added in a medium without extracellular calcium caused a discrete [Ca^2+^]_i_ rise. **(D) **Cimetidine (Cimet.) did not block the Ca^2+^ elevation caused by HA. Note that neither chlorpheniramine nor cimetidine modified basal Ca^2+ ^levels. **(E-P) **Representative images from the same experiment in **A **to **D **at different times: before the stimulus **(E, H, K, N)**, after HA addition **(F, I, L, O) **and 80 seconds after the stimulus was given **(G, J, M, P)**. Chlorpheniramine and cimetidine were incubated during the five minutes prior to HA addition. The bar on the side of image **E** indicates the scale for R = F_340 _/ F_380_. **(Q) **Average values of ratiometric Fura-2 measurements, expressed as a percentage of the basal (before stimulus) value are presented in the graph as the mean ± standard error from three to five independent experiments. ***P <0.0001 versus 100 μM HA and also versus 100 μM HA + Cimet.; ^*a*^*P *<0.01 versus 100 μM HA without extracellular Ca^2+^. HA, histamine; H_1_R, histamine receptor type 1; NPC, neural precursor cells.

To establish if addition of HA to NPC elicited increases in [Ca^2+^]_i_, P2 NPC growing with FGF-2 were loaded with the ratiometric calcium probe Fura-2 and re-plated in glass-bottom dishes for imaging. The intracellular calcium concentration is related to the ratio (R) of emission at 510 nm, resulting from alternating excitation of Fura-2 between 340 nm and 380 nm (R = F_340_/F_380_). We found that 65% of cells increased on average 2.2-fold the basal [Ca^2+^]_i_ in response to HA addition (Figure 3A). Such HA-elicited rise was completely blocked by co-incubation with chlorpheniramine (Figure 3B); cimetidine did not prevent the [Ca^2+^]_i_ increase produced by HA (Figure 3D). Antagonists added before HA did not modify the basal levels of [Ca^2+^]_i_ (Figures 3B and D). When experiments were done in the absence of extracellular Ca^2+^, we found a small but significant 1.3-fold increase in the fluorescence ratio stimulated by HA, caused by release of Ca^2+^ from intracellular deposits (Figure 3C). These results show that NPC express functional H_1_R, which after activation elicit a [Ca^2+^]_i_ increase.

### Histamine up-regulates transcripts for FGF receptors during proliferation

**Figure 4:**
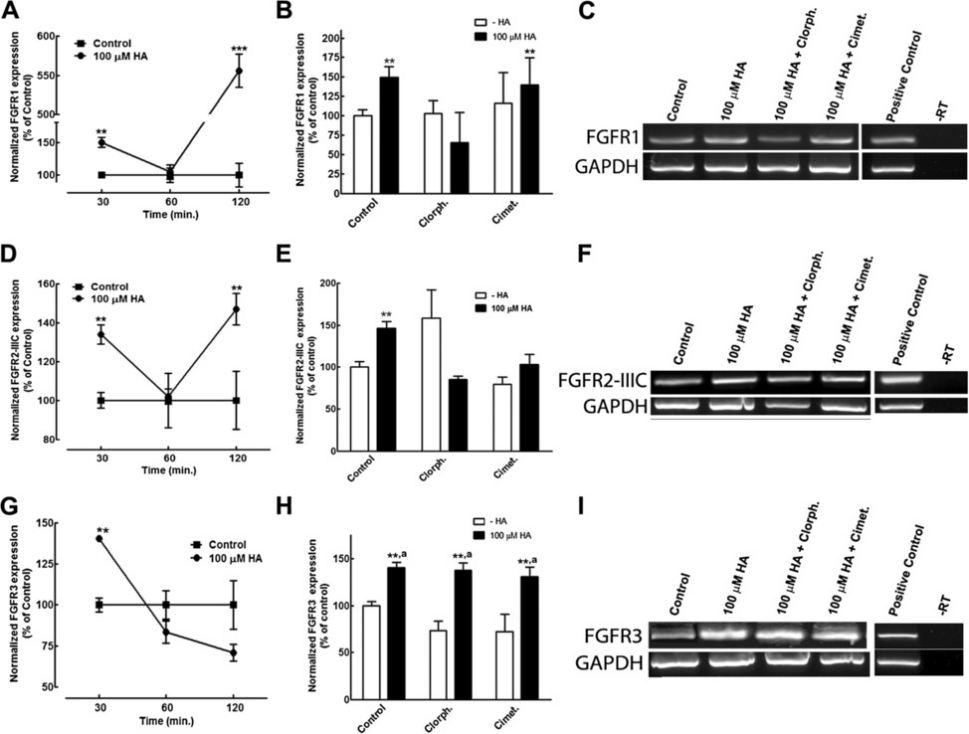
**HA up-regulates FGFRs transcripts. **RT-PCR analysis of the effect of 100 μM HA on FGFRs expression. Kinetics of the effect of HA on FGFR1 **(A)**, FGFR2-IIIC **(D) **and FGFR3 **(G) **expression, normalized by GAPDH; the increases are presented as percentage of the expression obtained in control conditions for each time point. **P <0.01 and ***P <0.001 versus control. **(B)** Pharmacological analysis of HA effect on FGFR1 expression at 30 minutes. The H_1_R antagonist chlorpheniramine (Chlorph.) prevented the increase in FGFR1 expression caused by HA, whereas cimetidine (Cimet.) was uneffective. **(E)** FGFR2-IIIC expression significantly increased after 120 minutes of HA treatment; both chlorpheniramine and cimetidine were able to block such increase. **(H) **HA increased FGFR3 transcripts, but such elevation was not sensitive to either chlorpheniramine or cimetidine. **P <0.01 versus control; ^*a*^*P* <0.01 versus its corresponding condition in the absence of HA. **(C, F and I)** Representative bands for the conditions shown in **B**, **E** and **H**, respectively, including a positive control (adult cerebral cortex) and PCR in the absence of retrotranscription (-RT). FGFRs, fibroblast growth factor receptors; HA, histamine.

In addition to calcium signaling, regulation of FGFRs might contribute to HA effects in neuronal commitment. We therefore analyzed the expression of FGFRs in proliferating NPC. In these experiments, cells were stimulated with HA and/or histaminergic drugs, and RNA was extracted at different time points (30, 60 or 120 minutes after treatment), followed by retrotranscription and endpoint PCR amplification with specific primers to detect FGFR1, FGFR2 (isoforms IIIB and IIIC), FGFR3 and FGFR4 expression. We were able to detect all listed FGFRs, except FGFR2-IIIB in control conditions. HA caused a transcriptional up-regulation of FGFR1, FGFR2-IIIC and FGFR3 in samples stimulated for 30 minutes. The expression of FGFR1 and FGFR2-IIIC showed a biphasic increase, while mRNA levels for FGFR3 showed a progressive decline after 60 to 120 minutes (Figures 4A, 4D and 4G).

The significant increase in mRNA for FGFR1 was 5.5 times relative to control after 2 hours of HA addition. FGFR1 up-regulation analyzed 30 minutes after HA stimulation, was sensitive to 1 μM chlorpheniramine, but not to 30 μM cimetidine, with neither antagonist having an effect on its own (Figures 4B and 4C). On the other hand, the HA-induced increase in FGFR2-IIIC mRNA showed a different pharmacology: at 30 minutes, neither antagonist had an effect, but after stimulation for 120 minutes, H_1_R or H_2_R antagonists were able to decrease FGFR2-IIIC expression to control levels (Figures 4E and 4F). FGFR3 expression was stimulated by HA treatment for 30 minutes; however, mRNA levels for this FGFR showed a progressive non-significant decline relative to control levels after 60 and 120 minutes (Figure 4G). At 30 minutes, the HA-elicited increase was neither prevented by chlorpheniramine nor by cimetidine (Figures 4H and 4I). Although we observed FGFR4 expression in control conditions, 100 μM HA, alone or in combination with H_1_R or H_2_R antagonists, did not significantly change such levels at the studied time points (data not shown).

### Histamine increases FOXP2 neurons after differentiation of cultured NPC

**Figure 5:**
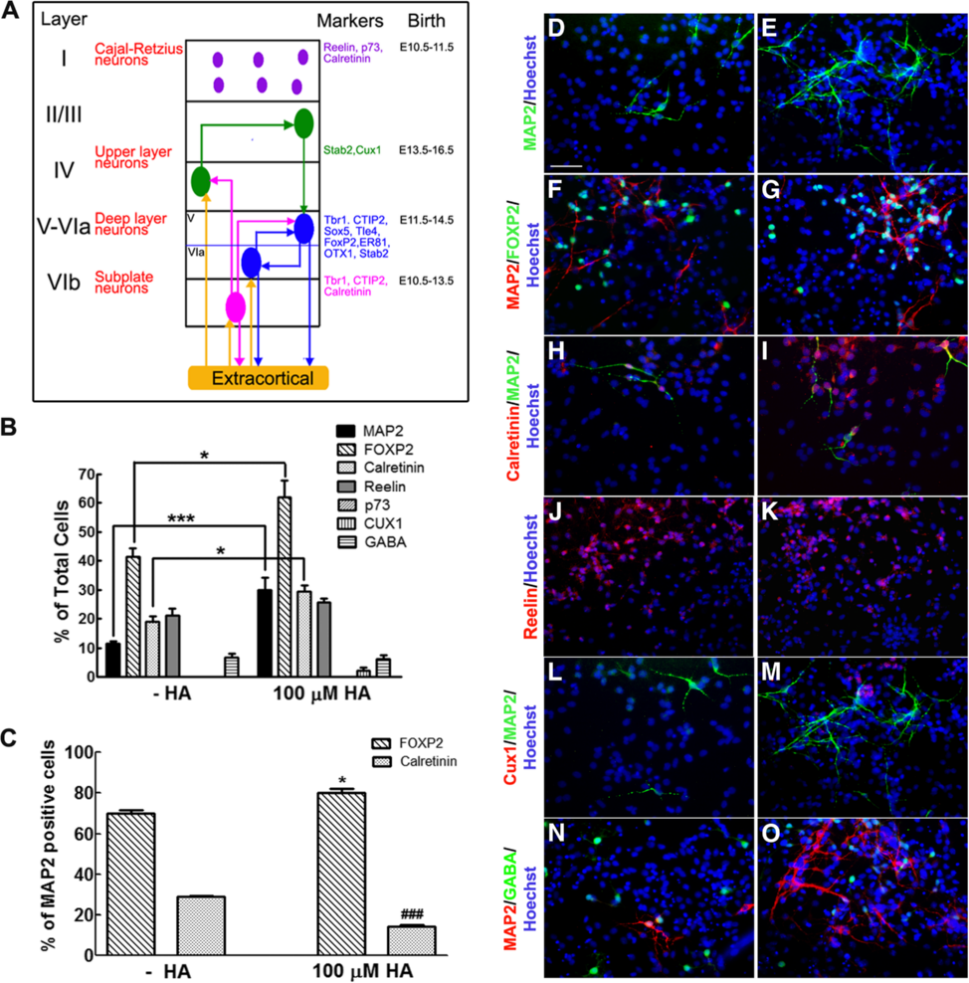
**HA induces differentiation of NPC to FOXP2 neurons. (A) **Modified scheme illustrating the expression of layer-specific markers in cortical neurons, their timing of generation *in vivo *and regions of projection [8,21,22]. **(B) **Quantification of the total number of mature neurons (MAP2+) and detection of specific cortical laminar neuronal phenotypes. *P <0.05 and ***P <0.001. **(C) **Percentage of MAP2-positive neurons that express FOXP2 or calretinin. Note that the proportion of FOXP2+ neurons increased whereas calretinin+ neurons decreased after HA treatment. *P <0.05 versus FOXP2 without HA; ^###^*P *<0.001 versus calretinin without HA. **(D-O)** Representative micrographs of immunofluorescence detection of the indicated markers in control **(D, F, H, J, L and N) **and HA-treated **(E, G, I, K, M and O) **cells. Nuclei were labeled with Hoechst and are shown in blue. Scale bar = 100 μm. HA, histamine; MAP2, microtubule associated protein 2; NPC, neural precursor cells.

In order to investigate if HA selectively induces specific neuronal phenotypes, we performed immunocytochemical detection of some markers characteristic of specific cortical layers after six days of differentiation. We used the following laminar markers (Figure 5A): p73 (Cajal-Retzius neurons), reelin (Cajal-Retzius neurons and subplate neurons), calretinin (Cajal-Retzius and subplate neurons), CUX1 (upper layer neurons) and FOXP2 (deeper layer neurons), together with the mature neuronal protein MAP2. As previously reported by our group, 100 μM HA caused a 2.7-fold increase in the number of MAP2+ cells (Figure 5B). We did not find, in control nor in HA-treated cultures, Cajal-Retzius neurons, since we did not observed any p73+ cells, although there were calretinin+ and reelin+ cells; such cells positive for calretinin and reelin might correspond to subplate neurons. Particularly the calretinin+ population was significantly increased (1.6-fold) in HA-treated cultures when quantified as cells expressing this marker relative to the total cell number (Figure 5B). However, given that the number of neurons also increased significantly after HA addition, we calculated the percentage of MAP2+ cells that also express calretinin. Surprisingly, HA caused a significant decrease in the proportion of calretinin+ neurons (Figure 5C). The molecular marker for the upper layer neurons CUX1 was not detected in control cultures, but CUX1+ cells were present in cultures treated with HA, although at a low proportion and with none of them co-expressing MAP2. HA did not affect the percentage of gamma-aminobutyric acid (GABA)+ cells after differentiation; these GABA-positive cells might be immature neurons, since they do not express MAP2.

A significant 1.5-fold increase was observed in the number of cells expressing the deep layer marker FOXP2 after HA addition, when quantified as the percent of total cells. This significant increase is also present when we analyzed the proportion of FOXP2+ cells that express MAP2 (Figure 5C), thus resulting in more neurons and a higher percentage of neurons that express FOXP2 after HA treatment.

### H_1_R antagonism prevents the induction of FOXP2 neuronal phenotype by histamine in cultured NPC and decreases the number of FOXP2 neurons in the cortical neuroepithelium *in vivo*

**Figure 6:**
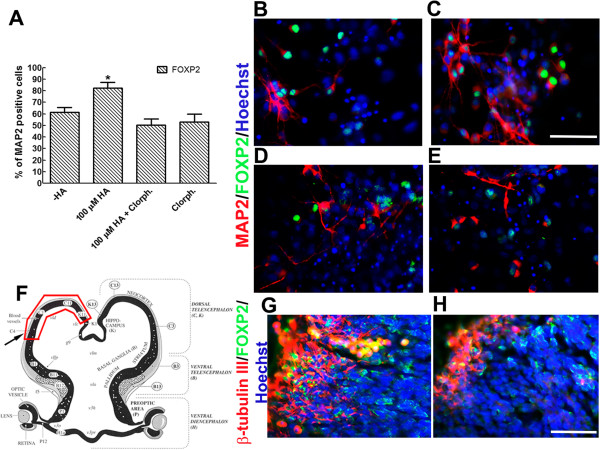
**Chlorpheniramine prevents FOXP2 neuronal differentiation i*n vitro *and *in vivo*. (A) **Significant increase of mature MAP2+ neurons positive for FOXP2 after HA stimulation of cultured NPC. This induction was blocked by co-incubation of HA with chlorpheniramine (Chlorph.). *P <0.01 versus all the remaining conditions. **(B to E) **Representative micrographs for control (-HA, **B**), 100 μM HA (**C**), 100 μM HA + 1 μM chlorpheniramine (**D**) and chlorpheniramine alone (**E**). **(F to H)** Effect of injection of chlorpheniramine in E12 developing rat brains that were analyzed two days later. **(F) **Scheme of a coronal section from an E14 rat telencephalon. The arrow represents the area used for analysis. The red line represents the section of the dorsal telencephalon used for *in vitro* experiments. **(G and H) **Representative images of coronal sections stained with antibodies for β-tubulin III and FOXP2. In control conditions (**G**), numerous cells are positive for these markers. In contrast, after injection of 25 μg of chlorpheniramine (**H**), a decrease in both β-tubulin III and FOXP2 was found. This effect was observed in four control and four chlorpheniramine-injected embryos from different dams. Scale bar = 100 μm. E, embryonic day; HA, histamine; MAP2, microtubule associated protein 2; NPC, neural precursor cells.

To investigate if the increase in the proportion of FOXP2+/MAP2+ cells caused by HA was sensitive to the H_1_R antagonism, we performed experiments in which co-incubation of HA and chlorpheniramine was made. This resulted in abolition of FOXP2 induction, with the antagonist alone having no effect relative to control conditions (Figures 6A to 6E). To study if activation of H_1_R might play a role during cortical development *in vivo*, we set up a system for intrauterine injection of E12 embryos in the lateral ventricles. Two days later, the dam was euthanized and the developing embryos recovered and analyzed by immunohistochemistry. In vehicle-injected animals, we found neurons expressing β-tubulin III that were also positive for FOXP2 (Figures 6F and 6G). However, after injection of 25 μg of chlorpheniramine, there was an apparent decrease in both β-tubulin III and FOXP2+ cells (Figure 6H), without obvious alterations in the cortical area. This effect seems to be specific for the developing cortex, because embryos that received the same amount of chlorpheniramine did not suffer a decrease in β-tubulin III staining in the midbrain (Escobedo-Ávila *et al*., unpublished observations).

## Discussion

NPC are useful to study the role of a number of factors affecting self-renewal, proliferation and cell fate *in vitro* and also can guide researchers on the compounds to be tested *in vivo*. We have previously reported, using cortical NPC, that HA increases cell division during the proliferative phase, in the presence of FGF-2, mainly through H_2_R activation without affecting the high proportion of Nestin-positive cells, a marker used for neural stem and precursor cells; after differentiation, this amine increases the proportion of neurons by H_1_R activation [16]. In this work, we show that: (i) HA, through H_1_R stimulation, decreases the area of clonal colonies and increases the proportion of colonies containing mature neurons after differentiation of NPC; (ii) HA promotes an increase in [Ca^2+^]_i_ by H_1_R activation in proliferating neural cells; (iii) HA receptor activation up-regulates FGFR1 and FGFR2-IIIC expression during proliferation; (iv) HA promotes differentiation of cultured NPC into FOXP2-positive neurons by H_1_R stimulation; and (v) blockade of H_1_R *in vivo* results in decreased numbers of FOXP2 neurons in the developing rat cerebral cortex.

To test if HA was able to induce neuronal commitment at the single cell level, we performed clonal analysis, the most stringent assay to test for differentiation potential *in vitro*. Whereas HA treatment produced a noticeable decrease in the size of colonies, incubation of HA+chlorpheniramine significantly increased the area of the colonies in relation to control and HA-treated cells. Cimetidine, on the other hand, did not antagonize HA action (Figure 1E). To investigate if these changes in colony area were related to variations in the total cell numbers per clone, this parameter was quantified. HA, chlorpheniramine and cimetidine, each added alone, significantly decreased cell numbers. The increase caused by HA was sensitive to chlorpheniramine, but not to cimetidine (Figure 1E). Images taken from the center of the colonies (Figures 1A to 1D) show that individual cells have similar sizes, but in the HA+ chlorpheniramine condition, cells were more disperse, suggesting that specific activation of H_2_R might promote migration/motility. On the other hand, H_1_R activation (HA+cimetidine) could be responsible for the compaction of the clonal colony. Further research is needed to elucidate these intriguing effects.

In clonal conditions, the differentiation potential of NPC shifts from neurogenic to astrocytogenic after several passages, similar to what happens in high-density cultures [23]. We show that HA is able to significantly increase neuronal differentiation, as measured by the proportion of colonies containing mature (MAP2+) neurons. The neuronal-promoting action of HA was also dependent upon activation of H_1_R, since this effect was sensitive to chlorpheniramine, but not to cimetidine. We also analyzed astrocytic differentiation in clonal cultures. Although at higher cell density, HA reduces the proportion of astrocytes [16], we found GFAP+ cells in all clonal colonies, indicating that neither HA nor its antagonists precluded astroglial commitment, and that the differentiation potential of a single NPC is conserved in the presence of HA. Therefore, HA seems to be promoting differentiation with neuronal bias in clonal cultures through activation of H_1_R.

Changes in [Ca^2+^]_i_ play diverse roles in nervous system development including differentiation of neural cells [24,25], chemotaxis [26,27], proliferation [28] and morphology [29]. Although a high proportion of NPC in our cultures expresses H_1_R (81%), only 65% express functional receptors, as shown by the increase in [Ca^2+^]_i_ promoted by HA. The increase in [Ca^2+^]_i_ due to HA was completely blocked by chlorpheniramine and significantly reduced in the absence of extracellular Ca^2+^. Activation of H_1_R leads to the production of IP_3_ and diacylglycerol, that in turn promote an increase in [Ca^2+^]_i_ due to activation of IP_3_ receptors in the endoplasmic reticulum, and activation of protein kinase C; our results following HA addition in the absence of Ca^2+^ in the extracellular medium strongly suggest that cultured NPC activate this intracellular-mediated Ca^2+^ increase. Calcium release from intracellular stores into the cytosol is a critical component during ontogenesis [19,20], and this HA-induced Ca^2+^ rise might contribute to neuronal commitment, with the involvement of several Ca^2+^-activated protein kinases and transcriptional regulation [30]. HA also increases [Ca^2+^]_i_ through H_1_R activation in undifferentiated mouse embryonic carcinoma pluripotent cells [18]. Recently, mouse embryonic stem cells were engineered to express a Ca^2+^-sensitive protein, and HA was used to select clones that augmented [Ca^2+^]_i_, confirming the presence of histaminergic G-protein coupled receptors in pluripotent stem cells. Furthermore, neural differentiation of these embryonic stem cells rendered cells that also were able to increase [Ca^2+^]_i_ after addition of 100 μM HA; all these effects were mediated by H_1_R [31], which is consistent with our findings.

The response of cells to FGFs during CNS development is mediated by membrane high-affinity FGFRs [32,33]. In particular, FGF-2 binds preferentially to FGFR1 and FGFR2, and with less affinity to FGFR3. FGF-2 knockout mice have only half of the neural precursor population when neurogenesis begins, leading to a loss of pyramidal neurons in the anterior cerebral cortex and to a dramatic decrease of glutamatergic cortical neurons in the adult brain [34-36]. In agreement, expression of a dominant negative form of FGFR1 in the dorsal telencephalic and mesencephalic areas from E9.5 onwards, revealed abnormalities in the medial prefrontal cortex and temporal cortical regions. A 30% to 40% reduction in cortical thickness which particularly affected the lower cortical layers was reported. This was accompanied by a reduction in cell number and neuronal soma size, shorter dendritic processes, as well as a marked reduction in the number of glutamatergic neurons from layer VI with no effects on GABAergic interneurons [37].

Regulation of FGFRs can participate in both proliferation and commitment of NPC. Our results using RT-PCR show that NPC express all FGFR tested, with the exception of FGFR2-IIIB, and that the expression of FGFR1 and FGFR2-IIIC were up-regulated at 30 and 120 minutes by HA with a transient decrease at 60 minutes. These changes might be explained by H_1_R desensitization, which occurs after 30 minutes and is relieved after 120 minutes in cerebral brain slices from adult rodents [38]. Previous studies demonstrated that *FGFR1* mRNA is expressed in the dorsal ventricular zone (VZ), the primordium of the cerebral cortex [39]. *FGFR2-IIIC* expression is particularly restricted to the VZ of the basal forebrain and other ventral structures where proliferation of precursors occurs [40]. FGFR3 and FGFR4 are strongly expressed in the VZ during CNS development, but at later stages, FGFR3 expression appears largely confined to glia [41,42]. We propose that HA stimulation of H_1_R regulates FGFR1 expression and these effects might be related to neuronal differentiation since the transcriptional increases of *FGFR1* were sensitive to H_1_R blockade, and *FGFR1* mRNA showed the highest increase (550% relative to control levels) after two hours of HA exposure.

It has been shown that [Ca^2+^]_i_ and FGFR activation are tightly related. FGF-2, but not epidermal growth factor, causes an increase in [Ca^2+^]_i_, which later on is correlated with neuronal differentiation in telencephalic neural precursors [43]. In addition, other factors might contribute to HA-induced neurogenesis. Our recent results [17] suggest that the HA neurogenic effect is at the level of cell commitment rather than a terminal differentiation effect: HA present only during differentiation of cortical NPC did not result in increased neuronal differentiation [17]. One of the underlying mechanisms for the neurogenic effect of HA includes increased expression of *Prox1* and *Neurogenin1* in the presence of FGF-2, which implies a HA-induced neuronal commitment. Accordingly, results reported recently indicate that HA induces neuronal differentiation from cultured mouse subventricular zone stem/progenitor cells by H_1_R activation, an effect that involves increased expression of *Mash1*, *Dlx2* and *Neurogenin1*[44]. Regarding differentiation of neurons in the cerebral cortex, several studies have implicated the participation of FGFR1 [37] and *Prox1* in neuron commitment and differentiation [45,46].

We found that HA, in addition to enhancing neuronal differentiation monitored through MAP2 expression, is also able to increase significantly the proportion of the neuronal population expressing the deep layer marker FOXP2 in cultured NPC. Calretinin, a marker that in principle is augmented after HA treatment when we analyzed the total number of cells, actually decreased when we calculated the proportion of calretinin+/MAP2+ cells before and after HA (Figure 5C). Thus, HA specifically caused increased numbers of FOXP2+ neurons. This induction was completely abolished by chlorpheniramine, identifying H_1_R as responsible for this effect.

The main source of cortical neurons is the neuroepithelium of the VZ, where precise timing generates first the deep layer (IV to VI) neurons (E11.5 to E14.5), followed by more superficial neurons (layers II/III), starting at E13.5 and ending at E16.5 (Figure 5A) [8,47]. HA concentration in the prosencephalon appears at E12 and peaks at E14 to E16 [9], suggesting a role of this biogenic amine on timing generation of specific types of neurons. Messenger RNA for histaminergic receptors are expressed at these stages of development as evidenced by *in situ* hybridization [48-50]. In particular, the cerebral cortex expresses H_1_R during development [48]. Intrauterine injection of chlorpheniramine in the lateral ventricles in E12 rat embryos precluded the expression of both β-tubulin III and FOXP2, suggesting a role of H_1_R during cerebral cortical development.

## Conclusions

In the present study we demonstrate that the neurogenic effect of HA by stimulation of H_1_R is present in clonal density cultures; we also show that H_1_R-induced neuronal differentiation correlates with an increase in [Ca^2+^]_i_ and the up-regulation of FGFR1. Interestingly, we found that 100 μM HA promotes differentiation of cerebrocortical NPC into FOXP2 neurons; in agreement, H_1_R antagonism *in vivo* interferes with β-tubulin III and FOXP2 immuno-reactivity in the developing cerebral cortex.

## Methods

### Cell culture

Animal procedures were approved by the local Animal Care and Use Committee and complied with local (NOM-062-ZOO-1999) and international guidelines (Animal Welfare Assurance A5281-01). In order to obtain multipotent NPC [51], E14 embryos were extracted from pregnant Wistar rats and cerebral cortices were dissected in Krebs solution (100 mM NaCl, 2 mM KCl, 0.6 mM KH_2_PO_4_, 12 mM NaHCO_3_, 2.2 mM MgSO_4_, 7 mM glucose, 0.1% phenol red and 0.3% bovine serum albumin). The tissue was mechanically dissociated to a single cell suspension. Cells were recovered by centrifugation, re-suspended and cultured on plasticware previously treated with 15 μg/mL poly-L-ornithine (Sigma, St. Louis, MO, USA) and 1 μg/ml human fibronectin (Invitrogen, Carlsbad, CA, USA) in fully defined N2 medium containing 10 ng/ml FGF-2 (Peprotech, Rocky Hill, NJ, USA) as mitogen. Passage (P) of cells was made with 0.1 mM ethylenediaminetetraacetic acid (EDTA) in PBS. P2 cells were maintained during eight (clonal) or four (high density) days in proliferative control (N2 medium + 10 ng/ml FGF-2) and experimental (N2 medium + 10 ng/mL FGF-2 + 100 μM HA alone and/or HR antagonist) conditions. Cells were seeded at low (1 x 10^3^ cells in 6-cm plates; clonal experiments), standard (1 x 10^4^ cells in 24-well plates for phenotype analysis) or high density (3 x 10^4^ in 6-well plates for mRNA expression analysis). Differentiation was promoted by removing FGF-2 and keeping the cells for six days in N2 medium + 200 μM ascorbic acid in the presence or absence of HA and/or HR antagonists. In both high-density and clonal experiments, HA and/or HR antagonists were present during both proliferation and differentiation stages.

HA H_1_R and H_2_R antagonists were used, in combination with 100 μM HA, to establish if such receptors were responsible for HA effects in clonal cultures, and to establish the participation of these histaminergic receptors on cell differentiation phenotypes. Chlorpheniramine (Sigma) was used as a H_1_R antagonist at 1 μM and as a H_2_R antagonist we used cimetidine at 30 μM (Sigma). H_1_R and/or H_2_R antagonists were added to control and 100 μM HA-treated cells during proliferation and differentiation phases. We also studied the effects of these antagonists on FGFRs expression at the end of proliferation phase.

For clonal analysis, reported procedures were followed [51]. In brief, P2 dissociated cells plated at 1,000 cells/6-cm dish were allowed to attach for three hours. Afterwards, 15 to 20 well-isolated cells were identified by a marking objective (Nikon, Tokyo, Japan) with a 1.8 mm circle. Only clones arising from single cells were analyzed. To make sure there was no contribution of migrating cells to the colony, the marked areas were monitored on a daily basis to detect cells coming close to the borders of the circle. A total of 5 to 7 clones in duplicate (10 to 14 colonies per experiment, n = 4 to 5) were analyzed.

### Immunocytochemistry and immunohistochemistry

Previously reported standard procedures were used [16,52]. Differentiated P2 cortical cells or tissue slices were fixed with 4% paraformaldehyde in PBS, pH 7.4 for 20 minutes at 4°C, permeabilized and blocked for 1 hour with 0.3% triton X-100 and 10% normal goat serum (NGS) in PBS. Samples were incubated overnight at 4°C with the following primary antibodies diluted in PBS containing 10% NGS: rabbit polyclonal anti-GFAP (1:2000, DAKO, Carpinteria, CA, USA); mouse monoclonal antibody anti-MAP2 (1:500, Millipore, Billerica, MA, USA); anti-β-tubulin III (1:2000, Covance, Princeton, NJ, USA); rabbit polyclonal anti-H_1_R (1:500, Santa Cruz Biotechnology, Dallas, TX, USA); goat polyclonal anti-H_2_R antibody (1:500, Santa Cruz Biotechnology); anti-Forkhead box 2, (FoxP2; 1:1000, Sigma); anti-reelin (1:600, Millipore); anti-Cut-like homeobox 1 (CUX1; 1:100, Sigma), anti-calretinin (1:1000, Swant, Marly, Switzerland), anti-tumor protein p73 (p73; 1:450, Santa Cruz Biotechnology), and anti-GABA (1:1000, Sigma). Alexa-Fluor 488 goat anti-rabbit immunoglobulin G (IgG) and Alexa 568 goat anti-mouse IgG were used as secondary antibodies (1:500; Invitrogen) diluted in PBS/10% NGS. Nuclei were stained with Hoechst 33258 (1 ng/ml; Sigma). Immunostainings were visualized and photographed with an epifluorescence microscope. Negative controls without primary antibodies did not show unspecific staining (data not shown).

### Cell counting

Cell counts from immunocytochemical stainings were performed in pictures taken with a Nikon digital camera and the Nikon ACT-1 imaging software. Quantification of cells was performed by counting the number of Hoechst stained nuclei (total cells) and the specified markers in at least eight random fields, from three to four independent experiments made in duplicate.

For clonal experiments, single cells were marked three hours after seeding and let to proliferate for eight days, followed by six days of differentiation. Immunodetection of MAP2 and GFAP was performed. Results are expressed as the percentage of colonies containing MAP2+ neurons, relative to total clonal colonies. In order to estimate the total number of cells in each clone, as well as to measure the area occupied by every colony, cultures were fixed in 10% formol-PBS, pH 7.4, washed and incubated with a 0.5% solution of crystal violet for 10 minutes at 21°C. After thorough washing with bi-distilled water, the number of cells from clonal colonies was counted, and the area of each clonal colony was measured from four to six experiments. For cell counting and area measurement, microphotographs were taken of single clones and merged in composite images with Adobe Photoshop software and analyzed with the aid of ImageJ software.

### Intracellular calcium measurements

To study changes in intracellular calcium ([Ca^2+^]_i_) induced by 100 μM HA, undifferentiated NPC were loaded for 40 minutes at 37°C in N2 medium with 5 μM Fura-2 AM, which diffuses across the cell membrane and is de-esterified by intracellular esterases to yield the ratiometric calcium probe Fura-2. Cells were detached, centrifuged and seeded at 1 x 10^5^ cells per 3.5-cm culture dishes with glass bottom (MatTek Corporation, Ashland, MA, USA), previously treated with 15 μg/ml poly-L-ornithine (Sigma) and 1 μg/ml human fibronectin (Invitrogen) in N2 medium. After 2 hours, attached cells were incubated at 37°C in Hank’s solution (137 mM NaCl, 5 mM KCl, 0.3 mM NaH_2_PO_4_, 0.8 mM MgSO_4_, 1 mM MgCl_2_, 5 mM CaCl_2_, 5 mM glucose, 2 mM glutamine, 10 mM Tris–HCl, pH 7.4) for 10 minutes; after this stabilization time, Hank’s solution was substituted by Hank’s with Ca^2+^ (5 mM) or without Ca^2+^, according to the experimental paradigm. Changes in [Ca^2+^]_i_ were monitored through ratiometric Fura-2 fluorescent signal after excitation alternating between 340 and 380 nm and emission set at 510 nm in a Carl Zeiss Live Cell Imaging system using AxioVision software at 37°C. Analysis of images was made offline by calculating the emission ratio generated by excitation at 340 nm divided by that measured at 380 nm (*R* = *F*_340_/*F*_380_). HR antagonists were added 5 minutes before HA stimulus and were maintained during HA stimulation.

### RNA extraction and RT-PCR

RNA was isolated from non-differentiated cultures using TRIZOL (Invitrogen). In order to have enough RNA, NPC were seeded at 3 x 10^4^ cells per well in 6-well plates, incubated with FGF-2 for four days, and treated at that point (time zero) with histaminergic compounds. Total RNA was extracted after 30, 60 or 120 minutes. RNA (1 μg) was reverse-transcribed with random hexamers and 500 to 800 ng from the RT reaction were used for PCR containing 2 U of Taq DNA polymerase (Invitrogen), 20 pmol of specific primers (Sigma; sequences listed below), 500 μM deoxynucleoside triphosphates and 1.5 mM MgCl_2_. Forward (F) and reverse (Rev) primer sequences (all in 5′-3′orientation) used for cDNA amplifications were as follows: FGFR1, F: CAACTggCTgCgggATgggg, Rev: gCgCCACAggCCTACggTTT (266 bp); FGFR2-IIIB, F: ggTCCTgAAgCACTCggggA, Rev: gCTAgCATCggggTgTCCgC (456 bp); FGFR2-IIIC, F: CCTgAAggCCgCCggTgTTA, Rev: CAgggggATgCgCTTggTCA (340 bp); FGFR3, F: gTgTggTgCCCTCTgACCgC, Rev: CACgCTgCCAgCCTCgTCAA (467 bp); FGFR4, F: CCCggCATCCCAgTggAggA, Rev: gTTggAgTCCCACggCCACg (392 bp); glyceraldehyde phosphate dehydrogenase (GAPDH), F: ATCACCATCTTCCAggAgCG, Rev: CCTgCTTCACCACCACCTTCTTg (573 bp).

For FGFR3 and FGFR4, amplification was made as follows: denaturalization at 95°C for 15 minutes, 30 cycles of denaturalization at 95°C for 1 minute, annealing at 62°C for 1 minute, and elongation at 72°C for 1 minute; for FGFR1 and FGFR2IIIC: denaturalization at 95°C for 15 minutes, 30 cycles of denaturalization at 95°C for 1 minute, annealing at 60°C for 1 minute, and elongation at 72°C for 1 minute; for FGFR2IIIB: denaturalization at 95°C for 15 minutes, 30 cycles of denaturalization at 95°C for 1 minute, annealing at 64°C for 1 minute, and elongation at 72°C for 1 minute. Final extension at 74°C for 10 minutes was terminated by rapid cooling at 4°C. Since we were not able to detect any FGFR2-IIIB expression, nested PCR reactions were performed, but we were still unable to see any amplification.

PCR products were analyzed in 2% agarose gel electrophoresis and the size of the reaction products was determined by comparison with molecular weight standards after ethidium bromide staining. Expression analysis was made by endpoint RT-PCR. Signals from the products obtained from the RT-PCR reactions were quantified by densitometry using ImageJ software (NIH). FGFRs expression was normalized to GAPDH expression assessed from the same cDNA in parallel PCR reactions and loaded in the same gels. The standardized optic density mean of each triplicated PCR was then expressed relative to the levels of GAPDH. As a negative control for PCR amplification, reactions with RNA in the absence of retro-transcription were included. The positive controls consisted of RNA extracted from adult cerebral cortex (FGFR1 and FGFR2-IIIC) or kidney (FGFR2-IIIB, FGFR3 and FGFR4), which were used to synthesize cDNA and amplified by PCR as described above.

Representative bands of the amplified PCR products were recovered from gels using the Qiaquick gel extraction kit (Qiagen, Germantown, MD, USA) according to the manufacturer’s instructions. These bands were sequenced at the Molecular Biology Unit in our Institute, and it was confirmed that all bands correspond indeed to FGFR.

### *In vivo* intrauterine injection of embryos

To study the effect of the H_1_R antagonist chlorpheniramine on cortical development *in vivo*, we used an ultrasound (Ultraview MHF-1) imaging device coupled to a microinjection system to introduce 2 μl of injectable water (control) or the same volume containing 25 μg of chlorpheniramine into the embryo’s telencephalic ventricles at E12 as described [53]. Briefly, the dam was placed in an airtight anesthesia chamber with 3% sevoflurane (Abbott Laboratories, Abbott Park, IL, USA) in 95% O_2_-5% CO_2_ gas mixture. The rats were maintained with a mask of inhaled anesthesia in 0.5% to 1.5% sevoflurane on a heating pad. The skin was shaved and aseptically prepared to make an incision in the abdominal skin and muscle. The uterine horns were exposed and a single embryo was secured to proceed with injection using a glass needle. The lateral ventricles were visualized while making the injections and embryos were identified by their position in the uterine horn. After 48 hours, the dam was euthanized and the E14 embryos were recovered. Only embryos that had a beating heart were selected for further analysis. After fixation with 4% paraformaldehyde in PBS (pH = 7.4) for 24 to 48 hours at 4°C, embryos were cryoprotected with 30% sucrose and rapidly frozen using liquid nitrogen. Coronal slices (20 μm) were obtained and stained with antibodies that recognize β-tubulin III or FOXP2.

### Statistics

All data are presented as mean ± standard error of the mean (S.E.M). One-way analysis of variance (ANOVA) was performed for statistical analysis, multiple comparisons were made using the post-hoc Student-Newman-Keuls test to compare data; for mRNA expression analysis we used the Dunnett test, in order to compare the experimental condition versus its corresponding control. Differences were considered statistically significant at *P* <0.05. Graphs and statistical analysis were performed using GraphPad and GraphPad Instat software respectively.

## Abbreviations

bp: base pair; CNS: central nervous system; E: embryonic day; F: forward; FGFR: fibroblast growth factor receptor; GABA: gamma-amino butyric acid; GADPH: glyceraldehyde phosphate dehydrogenase; GFAP: glial fibrillary acidic protein; HA: histamine; H1R: histamine type 1 receptor; H2R: histamine type 2 receptor; H3R: histamine type 3 receptor; IP3: inositol triphosphate; MAP2: microtubule associated protein 2; NGS: normal goat serum; NPC: neural precursor cells; P: passage; PBS: phosphate-buffered saline; R: ratio; Rev: reverse; RT-PCR: reverse transcriptase-polymerase chain reaction; VZ: ventricular zone.

## Competing interests

The authors declare that they have no competing interests.

## Authors’ contributions

AMH participated in all experiments and analyzed data. GRM contributed to clonal experiments and immunocytochemistry. IEA took part in intracellular calcium, RT-PCR measurements and intrauterine injections. IV contributed to experiment design/supervision and obtained funding. All authors participated in the preparation of the manuscript. All authors read and approved the final manuscript.

## Supplementary Material

Additional file 1: ** Proliferating NPC expresses H**_**1**_**R and H**_**2**_**R.** Description: Immunodetection of H_1_ (A and B), H_2_ (C and D) receptors (red) and nuclei stained with Hoechst (blue). The percentage indicates the proportion of cells expressing each receptor relative to total cells counted from 10 fields in control (A and C) and HA-treated (B and D) conditions. Note that 100 μM HA (B and D) does not modify the proportion of cells expressing histaminergic receptors. Scale bar = 100 μm. (TIFF 2322 kb) Click here for file
