# Supplementary figures and images for: Histamine up-regulates fibroblast growth factor receptor 1 and increases FOXP2 neurons in cultured neural precursors by histamine type 1 receptor activation: conceivable role of histamine in neurogenesis during cortical development in vivo
Source: Neural Dev. 2013 Mar 7;8:4. doi: 10.1186/1749-8104-8-4 (PMC3601999; doi:10.1186/1749-8104-8-4)

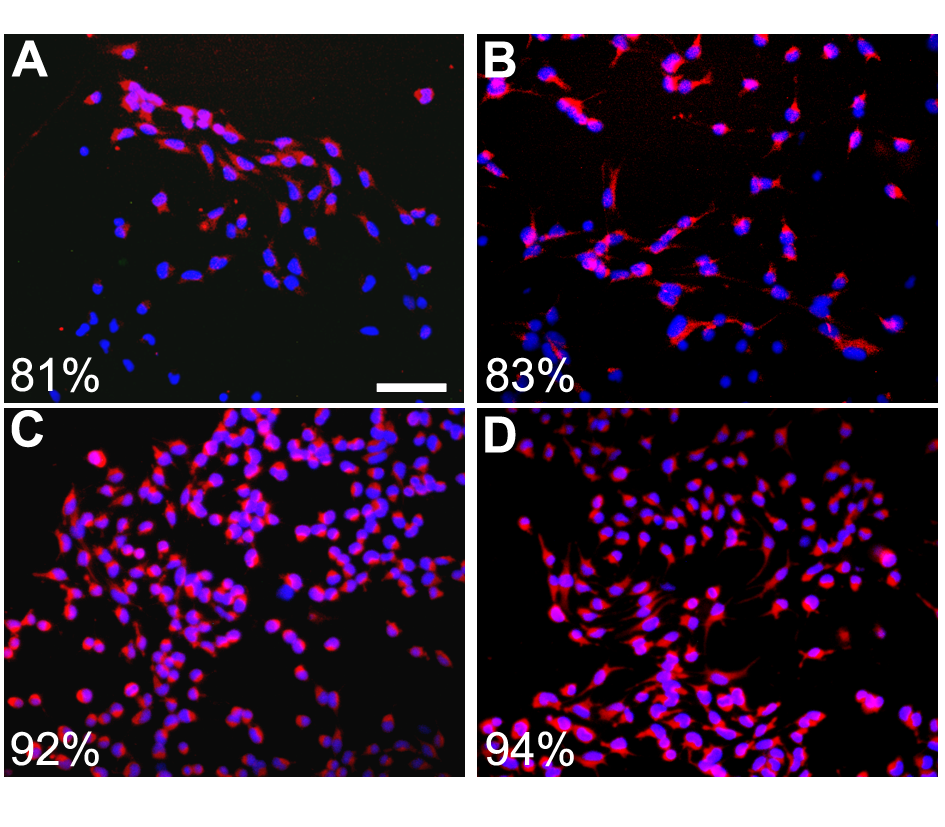

Supplement: Additional file 1: — Proliferating NPC expresses H1R and H2R. Description: Immunodetection of H1 (A and B), H2 (C and D) receptors (red) and nuclei stained with Hoechst (blue). The percentage indicates the proportion of cells expressing each receptor relative to total cells counted from 10 fields in control (A and C) and HA-treated (B and D) conditions. Note that 100 μM HA (B and D) does not modify the proportion of cells expressing histaminergic receptors. Scale bar = 100 μm. (TIFF 2322 kb) [file 1749-8104-8-4-S1.tiff]
